# Supplementary material for: Characterization of CD34+ Cells from Patients with Acute Myeloid Leukemia (AML) and Myelodysplastic Syndromes (MDS) Using a t-Distributed Stochastic Neighbor Embedding (t-SNE) Protocol
Source: Cancers (Basel). 2024 Mar 28;16(7):1320. doi: 10.3390/cancers16071320 (PMC11010974; doi:10.3390/cancers16071320)
Supplement: Supplementary file 1 [file cancers-16-01320-s001.zip › cancers-2918233-supplementary.pdf]

Supplementary Material to

Characterization of CD34[+] Cells from Patients with Acute Myeloid Leukemia (AML) and Myelodysplastic Syndromes (MDS) using a t-distributed stochastic neighbor embedding (t-SNE) protocol

Cathrin Nollmann, Wiebke Moskorz, Christian Wimmenauer, Paul S. Jäger, Ron P. Cadeddu, Jörg Timm, Thomas Heinzel and Rainer Haas

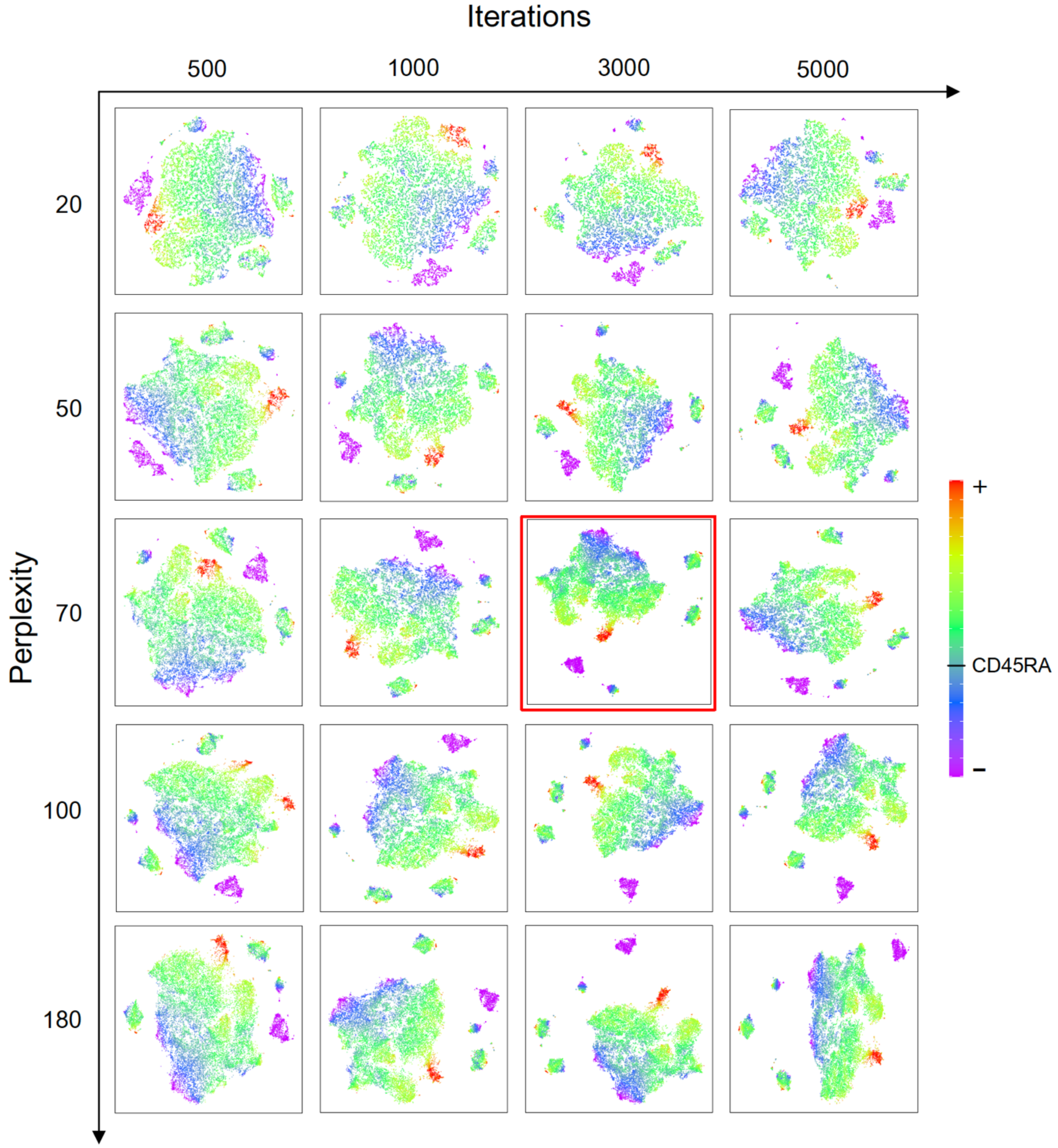

Figure S1. Evaluation of the impact of varring the t-SNE parameters number of iterations and perplexity on the t-SNE embedding. The whole dataset was analyzed. The parameter combination used for the analysis presented in the manuskript is framed in red. As an example, the expression of CD45RA in color code (color scale in arbitrary units).

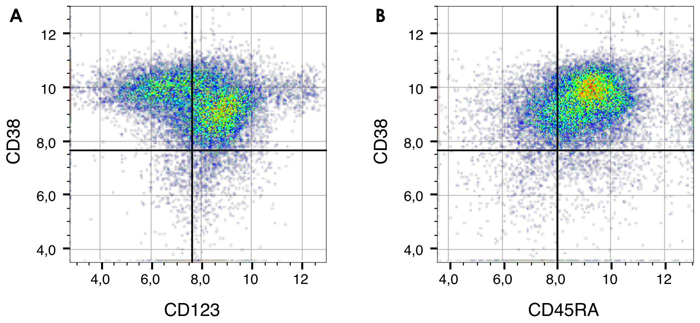

Figure S2. Scatter plots to determine the limits for the classification into *positive* and *negative* for the markers CD38 and CD123 (A) as well as for CD45RA (B). The fluorescence data was scaled biexponentially in a preliminary step.

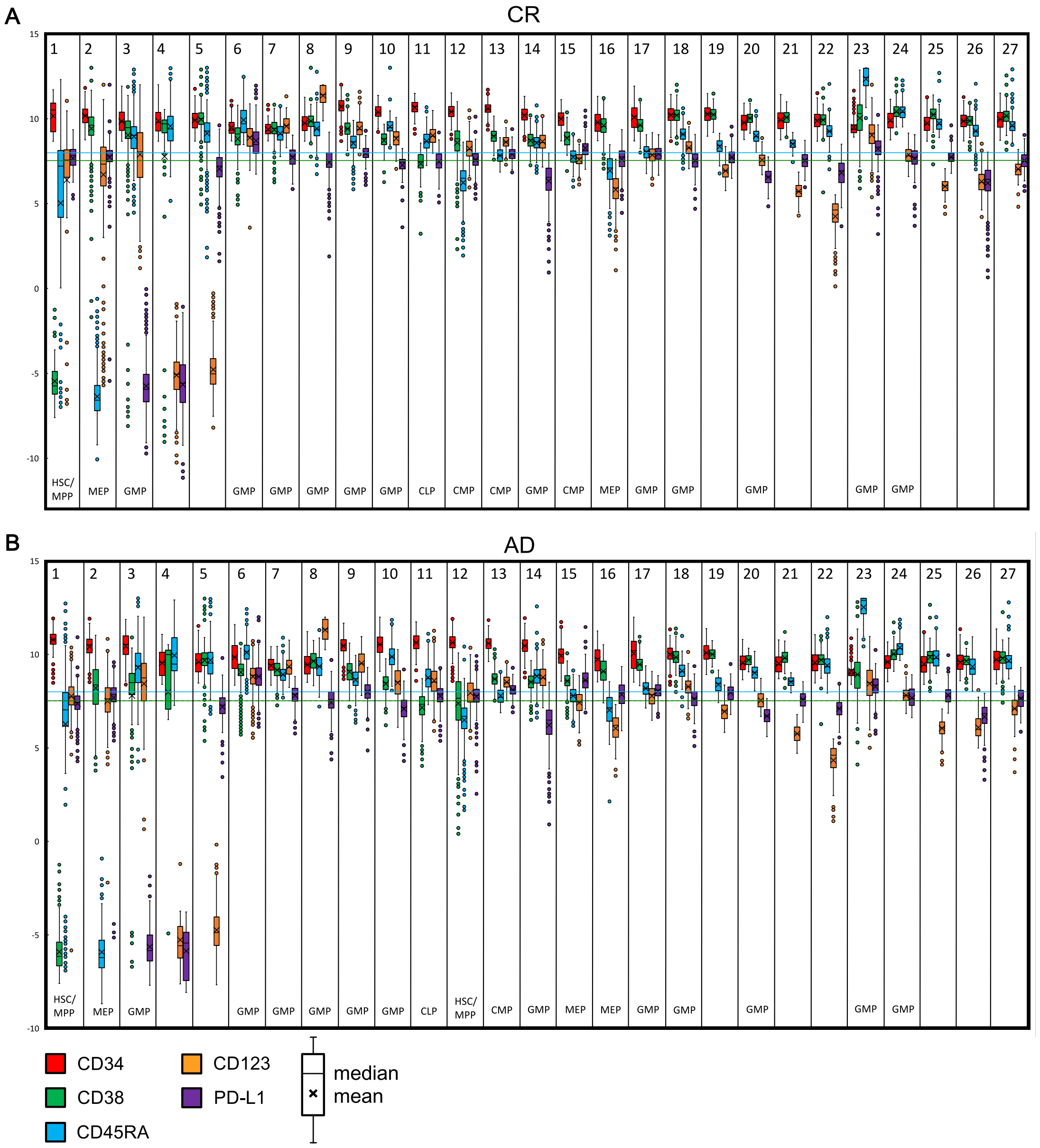

Figure S3. Box plots of the antigen expression levels of all 27 gates for (A) the patients in complete remission (CR) and (B) the patients with active disease (AD). The colored horizontal lines indicate the limits for the classification into *positive* and *negative* (blue line: CD45RA. green line: CD38 and CD123). The PD-L1 antigen was not used for the cell classification. The cell types (Table 2) assigned based on the fluorescence values are indicated at the bottom of the column: hematopoietic stem cells (HSC), multipotent progenitor cells (MPP), common lymphoid progenitors (CLP), common myeloid progenitors (CMP), megakaryocyte/ erythroid progenitors (MEP), granulocyte-macrophage progenitors (GMP).

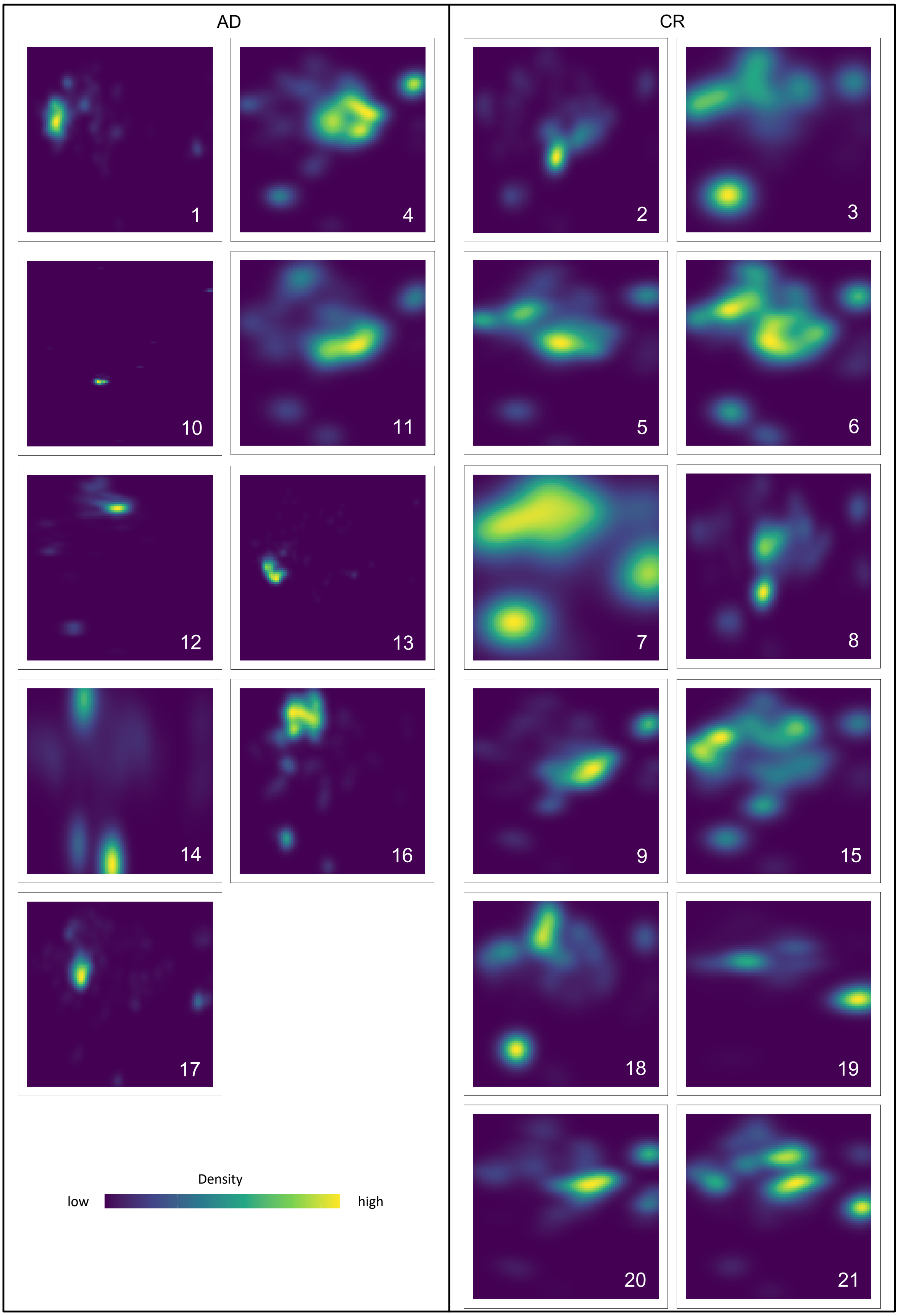

Figure S4. Density-plots of all patients, sorted according to active disease (AD, left) and complete remission (CR, right).

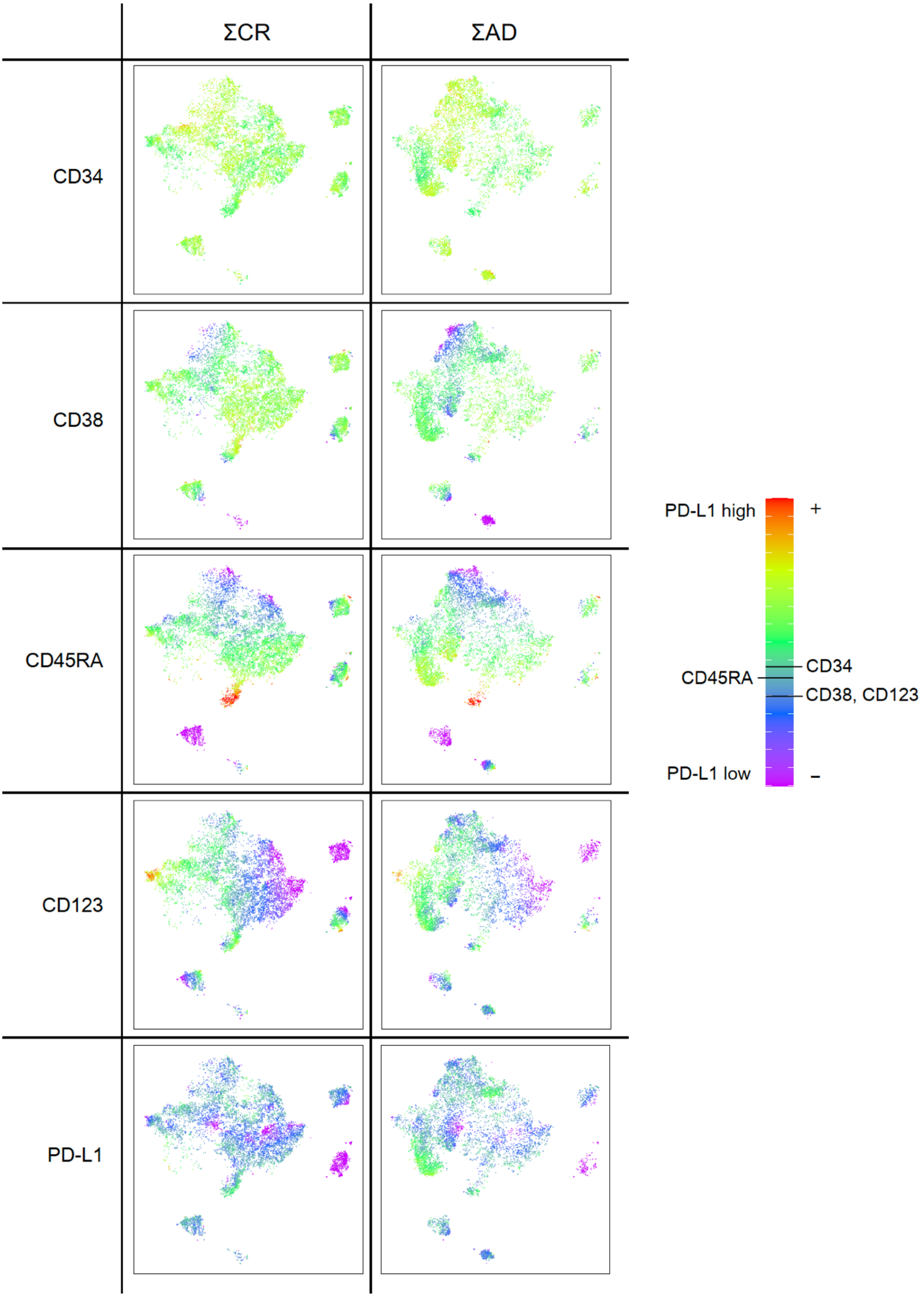

Figure S5. t-SNE represetations of the common datasets of patients in complete remission (CR) and of patients with active disease (AD)**.** The prevalence of the various markers entering the t-SNE algorithm are reproduced, for CD34, CD38, CD45RA, CD123 and PD-L1. The color scale represents the expression level in arbitrary units. The classification in terms of *positive* (+) and *negative* (-) expression is indicated by the black horizontal lines in the color bar.

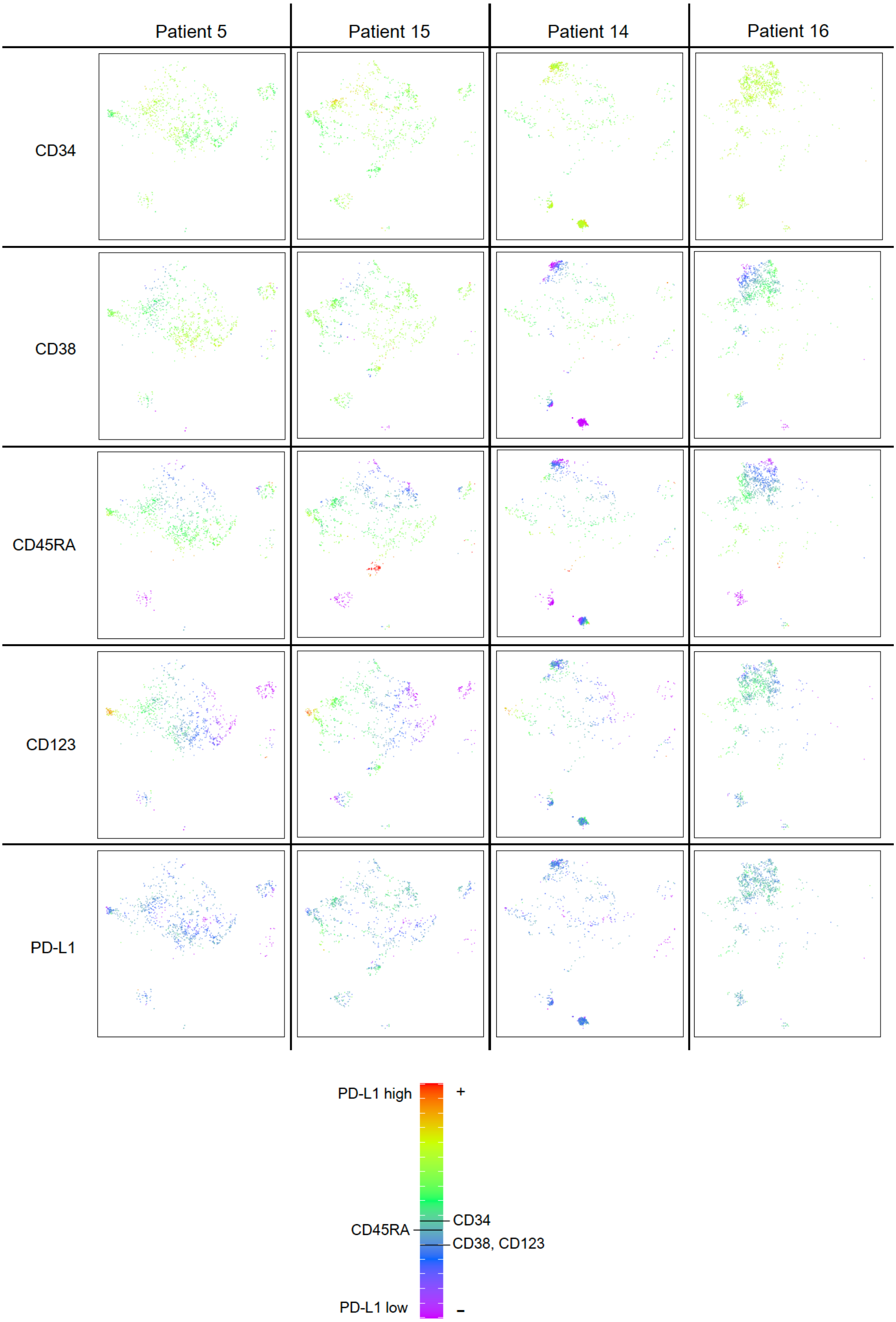

Figure S6. t-SNE represetations as an example for four patients, two each in complete remission (Patient 5, Patient 15) and with active disease (Patient 14, Patient 16). The prevalence of the various markers entering the t-SNE algorithm are reproduced, for CD34, CD38, CD45RA, CD123 and PD-L1. The color scale represents the expression level. The classification in terms of *positive* (+) and *negative* (-) expression is indicated by the black horizontal lines in the color bar.

Table S1. Percentage distribution of the cells in the 27 gates, defined in the t-SNE plot of the CR samples, for the three datasets (all patients, only AD, only CR).

Gate	All / [%]	AD / [%]	CR / [%]
1	2.50	0.65	4.79
2	5.17	5.93	4.23
3	4.64	6.88	1.87
4	0.49	0.79	0.12
5	4.86	6.35	3.01
6	9.33	1.42	19.13
7	3.37	2.87	4.00
8	2.48	3.59	1.12
9	4.10	4.98	3.00
10	2.70	0.84	5.01
11	2.49	1.22	4.06
12	6.85	4.69	9.52
13	4.10	3.60	4.73
14	3.75	3.84	3.63
15	3.93	1.15	7.36
16	3.97	5.48	2.10
17	2.71	3.43	1.82
18	5.31	7.14	3.04
19	2.59	3.51	1.45
20	2.02	2.65	1.23
21	1.57	1.83	1.26
22	3.38	4.39	2.13
23	3.57	4.71	2.16
24	1.72	2.30	1.01
25	2.91	3.20	2.55
26	5.27	7.53	2.49
27	4.22	5.05	3.18

Table S2. MRD analysis. All 12 patients in haematological CR were MRD negative. In two patients (7 and 9), leukaemia specific markers were monitored using next-generation sequencing (NGS) or fragment analysis, both yielding negative results. As our molecular biological methods do not detect any leukemia related mutation, MRD in the other patients is defined by a low WT1 level following an initially high expression or 100% donor chimerism.

Pat. ID	MRD markers
2	chimerism, ASXL1, WT1
3	chimerism, CEBPA, WT1
5	chimerism, WT1
6	chimerism, WT1
7	chimerism, NPM1
8	chimerism, WT1, JAK2, SF3B1
9	chimerism, CBFB-MYH11
15	chimerism, WT1, RUNX1, FISH
18	chimerism, WT1, FLT3-ITD
19	chimerism, ASXL1, WT1
20	chimerism, WT1, ASXL1, RUNX1
21	chimerism, WT1, ASXL1, RUNX1, TET2 and EZH2

Code S1. R script for the t-SNE analysis, calculation of the density matrices and some figures.

```
#LIBRARIES#############################################################
library(devtools)
library(flowCore)
library(flowClean)
library(flowClust)
library(Rtsne)
library(ggplot2)
install.packages("scales")
install.packages("ggthemes")
library(viridis)
library(png)
library(reshape2
library(MASS)
library(writexl)
library(dplyr)
############################################################

#Transformations
BiTrans <- biexponentialTransform(b = 1, d = 1)
LinTrans <- linearTransform(a = 1/100)

#Create Dataframe
df_t <- data.frame(FSCA=numeric(0), FSCH=numeric(0), FSCW=numeric(0),
SSCA=numeric(0), SSCH=numeric(0), SSCW=numeric(0), CD34=numeric(0),
CD38=numeric(0), Viability=numeric(0), CD123=numeric(0), CD45=numeric(0),
CD45RA=numeric(0),  PDL1=numeric(0), time=numeric(0), PatientID =
numeric(0), Control = numeric(0))

#Load files and transform
patients = c(1:21)
for (patient in patients) {
  setwd(toString(patient))
  fcm <- read.FCS(paste0("00",patient,".fcs" ))
  fcm.linear = transform(fcm,`FSC-A`=LinTrans(`FSC-A`),`FSC-
H`=LinTrans(`FSC-H`),`FSC-W`=LinTrans(`FSC-W`),`SSC-A`=LinTrans(`SSC-
A`),`SSC-H`=LinTrans(`SSC-H`),`SSC-W`=LinTrans(`SSC-W`),
                         `FJComp-APC-A`=BiTrans(`FJComp-APC-A`),
                         `FJComp-APC-Cy7-A`=BiTrans(`FJComp-APC-Cy7-A`),
```

```
                              `FJComp-AmCyan-A`=BiTrans(`FJComp-AmCyan-A`),
                              `FJComp-PE-A`=BiTrans(`FJComp-PE-A`),
                              `FJComp-PE-Cy5-A`=BiTrans(`FJComp-PE-Cy5-A`),
                              `FJComp-PE-Cy7-A`=BiTrans(`FJComp-PE-Cy7-A`),
                              `FJComp-PerCP-Cy5-5-A`=BiTrans(`FJComp-PerCP-Cy5-
5-A`) )
  df <- as.data.frame(fcm.linear@exprs)

  #Add parameter Patient
  df['Patient'] = patient

  #Add parameter control yes or no
  if(patient == 2 || patient == 3 || patient == 5 || patient == 6 ||
patient == 7 || patient == 8 || patient == 9 || patient == 15 || patient ==
18 || patient == 19 || patient == 20 || patient == 21){
    df['Control'] = 1 #Control
  } else {df['Control'] = 0} #Active Disease

  df_t <- rbind(df_t, df)
  setwd("..")
}


#Change column names
colnames(df_t) <- c("FSCA", "FSCH", "FSCW", "SSCA", "SSCH", "SSCW", "CD34",
"CD38", "Viability","CD123", "CD45", "CD45RA", "PDL1", "Time", "Patient",
"Control")

#Choose markers for t-SNE run
datamat_t <- as.matrix(df_t[c("CD34","CD38", "CD123", "CD45RA", "PDL1")])

#Run t-SNE for different parameters

iterations = c(500,1000,3000,5000)
perplexities = c(20,50,70,100,180)
markers = c("CD34", "CD38", "CD123", "CD45RA", "PDL1")

for (iteration in iterations){
  for(perp in perplexities){
    tsne <- Rtsne(datamat_t, dims = 2, perplexity=perp, verbose=TRUE,
max_iter = iteration, check_duplicates = FALSE)
    df_t[c('tSNE1', 'tSNE2')] <- as.data.frame(tsne$Y)
    perplexity <- perp
    dataname <- paste0("Data_tSNE_it",iteration,"_p",perp,".Rdata")
    save(data, file = dataname)
      for (marker in markers){
        ggplot(data=subset(df_t, ), aes_string(x="tSNE1", y="tSNE2",colour
= `marker`)) +geom_point(size = 0.1) +
scale_colour_gradientn(colours=rev(rainbow(5)), limits = c(5, 13), oob =
scales::squish) + theme_linedraw() + theme(panel.grid = element_blank(),
axis.text = element_blank(), axis.title = element_blank(), axis.ticks =
element_blank()) + guides(color = FALSE)
        ggsave(paste0(marker,"_p",perp, ".pdf"), plot = last_plot(),
               scale = 1, width = 20, height = 20, dpi = 600,
               units = "cm")
      }
  }
}
```

```
#Create scatter plots
#Note that you have to adjust the limits according to your transformation

markers = c("CD34", "CD38", "CD123", "CD45RA", "PDL1")

#Common scatter plot all Patients, fluorescent markers color-coded
for (marker in markers){
  ggplot(data=subset(df_t,), aes_string(x="tSNE1", y="tSNE2",colour =
`marker`)) +geom_point(size = 0.1)  +
scale_colour_gradientn(colours=rev(rainbow(5)), limits = c(5, 13), oob =
scales::squish) + theme_linedraw() + theme(panel.grid = element_blank())
  ggsave(paste0(marker,"_p",perplexity, ".pdf"), plot = last_plot(),
         scale = 1, width = 22, height = 20, dpi = 600,
         units = "cm")
}

#Common scatter plot all patients with active disease, fluorescent markers
color-coded
for (marker in markers){
  ggplot(data=subset(df_t, Control==0), aes_string(x="tSNE1",
y="tSNE2",colour = `marker`)) +geom_point(size = 0.1) + theme_classic() +
scale_colour_gradientn(colours=rev(rainbow(5)), limits = c(5, 13), oob =
scales::squish) + theme_linedraw() + theme(panel.grid = element_blank())
  ggsave(paste0(marker,"_p",perplexity, "_AD", ".pdf"), plot = last_plot(),
         scale = 1, width = 22, height = 20, dpi = 600,
         units = "cm")
}

#Common scatter plot all control patients, fluorescent markers color-coded
for (marker in markers){
  ggplot(data=subset(df_t, Control==1), aes_string(x="tSNE1",
y="tSNE2",colour = `marker`)) +geom_point(size = 0.1) + theme_classic() +
scale_colour_gradientn(colours=rev(rainbow(5)), limits = c(5, 13), oob =
scales::squish) + theme_linedraw() + theme(panel.grid = element_blank())
  ggsave(paste0(marker,"_p",perplexity,"_CR", ".pdf"), plot = last_plot(),
         scale = 1, width = 22, height = 20, dpi = 600,
         units = "cm")
}

#Scatter plot for every patient, fluorescent markers color-coded
for (marker in markers){
  for (patient in patients){
    ggplot(data=subset(df_t,Patient == patient), aes_string(x="tSNE1",
y="tSNE2",colour = `marker`)) +geom_point(size = 0.1)  +
scale_colour_gradientn(colours=rev(rainbow(5)), limits = c(5, 13), oob =
scales::squish) + theme_linedraw() + theme(panel.grid = element_blank())
    ggsave(paste0("Pat",patient,"_", marker, "_p",perplexity,".pdf"), plot
= last_plot(),
           scale = 1, width = 22, height = 20, dpi = 600,
           units = "cm")
  }
}

#Create density plots with overlay of the scatter plot

#Common density plot all patients
ggplot(data=subset(df_t), aes(x=tSNE1, y=tSNE2)) +
scale_x_continuous(limits =range(df_t$tSNE1)) + scale_y_continuous(limits =
range(df_t$tSNE2) )  +geom_point(size = 0.1) +
geom_density2d_filled(contour_var = "ndensity", alpha = 0.7 ) +
theme_linedraw() + theme(panel.grid = element_blank())
ggsave(paste0("Densityplot_Scatter_p",perplexity,".pdf"), plot =
last_plot(),
       scale = 1, width = 22, height = 20, dpi = 600,
```

```
       units = "cm")

#Common density plot with overlay of the scatter plot patients with active
disease
ggplot(data=subset(df_t, Control == 0), aes(x=tSNE1, y=tSNE2)) +
scale_x_continuous(limits =range(df_t$tSNE1)) + scale_y_continuous(limits =
range(df_t$tSNE2) )  +geom_point(size = 0.1) +
geom_density2d_filled(contour_var = "ndensity",alpha = 0.7) +
theme_linedraw() + theme(panel.grid = element_blank())
ggsave(paste0("Densityplot_Scatter_AD_p",perplexity,".pdf"), plot =
last_plot(),
       scale = 1, width = 22, height = 20, dpi = 600,
       units = "cm")

#Common density plot with overlay of the Scatter plot patients control
ggplot(data=subset(df_t, Control == 1), aes(x=tSNE1, y=tSNE2)) +
scale_x_continuous(limits =range(df_t$tSNE1)) + scale_y_continuous(limits =
range(df_t$tSNE2) )  +geom_point(size = 0.1) +
geom_density2d_filled(contour_var = "ndensity",alpha = 0.7) +
theme_linedraw() + theme(panel.grid = element_blank())
ggsave(paste0("Densityplot_Scatter_CR_p",perplexity,".pdf"), plot =
last_plot(),
      scale = 1, width = 25, height = 22, dpi = 600,
      units = "cm")


#Calculate and plot density matrix
#For every single patient

for (patient in patients) {

  data_sub = subset(df_t, Patient == patient )
  density_matrix_list = kde2d(data_sub$tSNE1, data_sub$tSNE2,lims =
c(range(df_t$tSNE1), range(df_t$tSNE2)), n=90)

  rownames(density_matrix_list$z) = density_matrix_list$x
  colnames(density_matrix_list$z) = density_matrix_list$y

  density_matrix= melt(density_matrix_list$z,
id.var=rownames(density_matrix_list))
  names(density_matrix) = c("tSNE1","tSNE2","z")

  write_xlsx(density_matrix,
paste0('Density_matrix_patient',patient,'.xlsx'))

  ggplot(density_matrix, aes(tSNE1, tSNE2, z=z, fill=z)) + geom_tile() +
scale_fill_viridis_c() + theme_linedraw() + theme(panel.grid =
element_blank())
  ggsave(paste0( "Densitymatrix_patient_",patient, ".png"), plot =
last_plot(),
         scale = 1, width = 25, height = 20, dpi = 600,
         units = "cm")

}

#Common density matrix for patient groups
patients_AD = c(1,4,10,11,12,13,14,16,17)
patients_control = c(2,3,5,6,7,8,9,15,18,19,20,21)

#Common density matrix patients with active disease

data_sub = subset(df_t, Control == 0)

density_matrix_list = kde2d(data_sub$tSNE1, data_sub$tSNE2,lims =
c(range(df_t$tSNE1), range(df_t$tSNE2)), n=90)
```

```
rownames(density_matrix_list$z) = density_matrix_list$x
colnames(density_matrix_list$z) = density_matrix_list$y


density_matrix= melt(density_matrix_list$z,
id.var=rownames(density_matrix_list))
names(density_matrix) = c("tSNE1","tSNE2","z")

write_xlsx(density_matrix, paste0('Density_matrix_AD.xlsx'))

ggplot(density_matrix, aes(tSNE1, tSNE2, z=z, fill=z)) + geom_tile() +
scale_fill_viridis_c() + theme_linedraw() + theme(panel.grid =
element_blank())
ggsave(paste0( "Densitymatrix_AD.pdf"), plot = last_plot(),
       scale = 1, width = 22, height = 20, dpi = 600,
       units = "cm")


#Common density matrix patients with active disease without patient k
for(k in patients_AD){

  data_sub = subset(df_t, Control == 0 & Patient != k )

  density_matrix_list = kde2d(data_sub$tSNE1, data_sub$tSNE2,lims =
c(range(df_t$tSNE1), range(df_t$tSNE2)), n=90)

  rownames(density_matrix_list$z) = density_matrix_list$x
  colnames(density_matrix_list$z) = density_matrix_list$y


  density_matrix= melt(density_matrix_list$z,
id.var=rownames(density_matrix_list))
  names(density_matrix) = c("tSNE1","tSNE2","z")

  write_xlsx(density_matrix, paste0('Density_matrix_AD_woPat',k,'.xlsx'))

  ggplot(density_matrix, aes(tSNE1, tSNE2, z=z, fill=z)) + geom_tile() +
scale_fill_viridis_c() + theme_linedraw() + theme(panel.grid =
element_blank())
  ggsave(paste0( "Densitymatrix_AD_woPat",k,".pdf"), plot = last_plot(),
         scale = 1, width = 22, height = 20, dpi = 600,
         units = "cm")
}

#Common density matrix patients with active disease without patient 4 and
11

  data_sub = subset(df_t, Control == 0 & Patient != 4 & Patient != 11 )

  density_matrix_list = kde2d(data_sub$tSNE1, data_sub$tSNE2,lims =
c(range(df_t$tSNE1), range(df_t$tSNE2)), n=90)

  rownames(density_matrix_list$z) = density_matrix_list$x
  colnames(density_matrix_list$z) = density_matrix_list$y


  density_matrix= melt(density_matrix_list$z,
id.var=rownames(density_matrix_list))
  names(density_matrix) = c("tSNE1","tSNE2","z")

  write_xlsx(density_matrix,
paste0('Density_matrix_AD_woPat4_woPat11.xlsx'))

  ggplot(density_matrix, aes(tSNE1, tSNE2, z=z, fill=z)) + geom_tile() +
```

```
scale_fill_viridis_c() + theme_linedraw() + theme(panel.grid =
element_blank())
  ggsave(paste0( "Densitymatrix_AD_woPat4_woPat11.pdf"), plot =
last_plot(),
         scale = 1, width = 22, height = 20, dpi = 600,
         units = "cm")

#Common density matrix patients with active disease without patient 4, 11
and patient k

  data_sub = subset(df_t, Control == 0 & Patient != 4 & Patient != 11 &
Patient != k  )

  density_matrix_list = kde2d(data_sub$tSNE1, data_sub$tSNE2,lims =
c(range(df_t$tSNE1), range(df_t$tSNE2)), n=90)

  rownames(density_matrix_list$z) = density_matrix_list$x
  colnames(density_matrix_list$z) = density_matrix_list$y


  density_matrix= melt(density_matrix_list$z,
id.var=rownames(density_matrix_list))
  names(density_matrix) = c("tSNE1","tSNE2","z")

  write_xlsx(density_matrix,
paste0('Density_matrix_AD_woPat4_woPat11_woPat',k,'.xlsx'))

  ggplot(density_matrix, aes(tSNE1, tSNE2, z=z, fill=z)) + geom_tile() +
scale_fill_viridis_c() + theme_linedraw() + theme(panel.grid =
element_blank())
  ggsave(paste0( "Densitymatrix_AD_woPat4_woPat11_woPat",k,".pdf"), plot =
last_plot(),
         scale = 1, width = 22, height = 20, dpi = 600,
         units = "cm")


#Common density matrix patients control

data_sub = subset(df_t, Control == 1)

density_matrix_list = kde2d(data_sub$tSNE1, data_sub$tSNE2,lims =
c(range(df_t$tSNE1), range(df_t$tSNE2)), n=90)

rownames(density_matrix_list$z) = density_matrix_list$x
colnames(density_matrix_list$z) = density_matrix_list$y


density_matrix= melt(density_matrix_list$z,
id.var=rownames(density_matrix_list))
names(density_matrix) = c("tSNE1","tSNE2","z")

write_xlsx(density_matrix, paste0('Density_matrix_CR.xlsx'))

ggplot(density_matrix, aes(tSNE1, tSNE2, z=z, fill=z)) + geom_tile() +
scale_fill_viridis_c()  + theme_linedraw() + theme(panel.grid =
element_blank())
ggsave(paste0( "Densitymatrix_CR.pdf"), plot = last_plot(),
       scale = 1, width = 22, height = 20, dpi = 600,
       units = "cm")


#Common density matrix patients control without patient k

for(k in patients_control){
```

```
  data_sub = subset(df_t, Control == 1 & Patient != k )

  density_matrix_list = kde2d(data_sub$tSNE1, data_sub$tSNE2,lims =
c(range(df_t$tSNE1), range(df_t$tSNE2)), n=90)

  rownames(density_matrix_list$z) = density_matrix_list$x
  colnames(density_matrix_list$z) = density_matrix_list$y


  density_matrix= melt(density_matrix_list$z,
id.var=rownames(density_matrix_list))
  names(density_matrix) = c("tSNE1","tSNE2","z")

  write_xlsx(density_matrix, paste0('Density_matrix_CR_woPat',k,'.xlsx'))

  ggplot(density_matrix, aes(tSNE1, tSNE2, z=z, fill=z)) + geom_tile() +
scale_fill_viridis_c()  + theme_linedraw() + theme(panel.grid =
element_blank())
  ggsave(paste0( "Densitymatrix_CR_woPat",k,".pdf"), plot = last_plot(),
         scale = 1, width = 22, height = 20, dpi = 600,
         units = "cm")
}


#Convert dataframe in CSV file

#For all patients

datasub_csv <- subset(df_t)
write.csv(datasub_csv, paste0("csv_dataframe_all.csv"), row.names=FALSE)


#For every single patient

for(patient in patients){
  datasub_csv <- subset(df_t, Patient==patient)
  write.csv(datasub_csv, paste0("csv_dataframe_",patient,".csv"),
row.names=FALSE)
}

#All patients with active disease

datasub_csv <- subset(df_t, Control ==0)
write.csv(datasub_csv, paste0("csv_dataframe_AD.csv"), row.names=FALSE)

#All patients control

datasub_csv <- subset(df_t, Control ==1)
write.csv(datasub_csv, paste0("csv_dataframe_control.csv"),
row.names=FALSE)


#Read FSC File of a single FlowJo Gate and convert it into a CSV File

for(k in 1:27) {
  fcm_temp <- read.FCS(paste0("export_csv_dataframe_all_",k,".fcs" ),
truncate_max_range = TRUE)
  data_csv_temp <- as.data.frame(fcm_temp@exprs)
  colnames(data_csv_temp) <- c("FSCA",
"Viability","CD123","CD45","CD45RA","PDL1","FSCH","Time","Patient","tSNE1",
"tSNE2","FSCW","SSCA","SSCH","SSCW","CD34","CD38")
  data_csv_temp <- data_csv_temp[c(paste0("FSCA"), paste0("FSCH"),
paste0("FSCW"), paste0("SSCA"), paste0("SSCH"), paste0("SSCW"),"CD45",
"CD34", "CD38", "CD45RA", "CD123", "PDL1", "Time", "Patient", "tSNE1",
"tSNE2")]
```

```
  write.csv(data_csv_temp, paste0("csv_dataframe_all_gate_",k,".csv"),
row.names=FALSE)
}
```

Code S2. Python script for quantitative analysis of the t-SNE plots with the Pearson coefficient

```
import numpy as np
import pandas as pd


#Calculate Pearson  Coefficient

def pearson_coeff(density1, density2):
    df = pd.concat([density1[density1.columns[-1]],
                    density2[density2.columns[-1]]], axis=1)
    df.columns = ['d1', 'd2']
    pearson = df.cov().iloc[1, 0] / (df.iloc[:, 0].std() * df.iloc[:,
1].std())
    return(pearson)


#Compare common dataset AD vs common dataset control

datasets = []

datasets.append(pd.read_excel("Density_matrix_AD.xlsx"))
datasets.append(pd.read_excel("Density_matrix_CR.xlsx"))

R = []

for df1 in datasets:
    tempR = []

    for df2 in datasets:
        tempR.append(pearson_coeff(df1, df2))
    R.append(tempR)

R = np.array(R)


#Compare common dataset AD / common dataset Control with single patients
NAD / NControl

dataset_AD_woN = []
dataset_control_woN = []
dataset_NAD = []
dataset_NAD_woPat4Pat11 = []
dataset_Ncontrol = []
dataset_control = []
dataset_AD = []
dataset_AD_woPat4_Pat11 = []
dataset_AD_woPat4_Pat11_woN = []

patients_AD = [1, 4, 10, 11, 12, 13, 14, 16, 17]
patients_control = [2, 3, 5, 6, 7, 8, 9, 15, 18, 19, 20, 21]
patients_AD_woPat4Pat11 = [1, 10, 12, 13, 14, 16, 17]

#Compare common dataset AD without patient N vs single patient NAD

R_AD = []

for j in range(9):
```

```
    dataset_AD_woN.append(pd.read_excel(
        "Density_matrix_AD_woPat{}.xlsx".format(patients_AD[j])))
    dataset_NAD.append(pd.read_excel(
        "Density_matrix_patient{}.xlsx".format(patients_AD[j])))
    tempR = []
    tempR.append(pearson_coeff(dataset_AD_woN[j], dataset_NAD[j]))
    R_AD.append(tempR)


R_AD = np.array(R_AD)

np.savetxt('pearson_coefficient_AD.csv', R_AD, delimiter=',')


#Compare common dataset control without patient N vs single patient
Ncontrol

R_control = []

for k in range(12):

    dataset_control_woN.append(pd.read_excel(
        "Density_matrix_CR_woPat{}.xlsx".format(patients_control[k])))
    dataset_Ncontrol.append(pd.read_excel(
        "Density_matrix_patient{}.xlsx".format(patients_control[k])))

    tempR = []
    tempR.append(pearson_coeff(dataset_control_woN[k],
dataset_Ncontrol[k]))
    R_control.append(tempR)

R_control = np.array(R_control)

np.savetxt('pearson_coefficient_control.csv', R_control, delimiter=',')


#Compare common dataset control vs single patient NAD

R_NAD_vs_control = []

dataset_control.append(
    pd.read_excel("Density_matrix_CR.xlsx"))

for j in range(9):

    tempR = []
    tempR.append(pearson_coeff(
        dataset_control[0], dataset_NAD[j]))
    R_NAD_vs_control.append(tempR)

R_NAD_vs_control = np.array(R_NAD_vs_control)

np.savetxt('pearson_coefficient_NAD_vs_control.csv',
           R_NAD_vs_control, delimiter=',')



#Compare common dataset AD vs single patient N Control

R_Ncontrol_vs_AD = []

dataset_AD.append(pd.read_excel(
    "Density_matrix_AD.xlsx"))
```

```
for i in range(12):

    tempR = []
    tempR.append(pearson_coeff(
        dataset_AD[0], dataset_Ncontrol[i]))
    R_Ncontrol_vs_AD.append(tempR)


R_Ncontrol_vs_AD = np.array(R_Ncontrol_vs_AD)

np.savetxt('pearson_coefficient_Ncontrol_vs_AD.csv',
           R_Ncontrol_vs_AD, delimiter=',')


#Compare common dataset AD without Pat 4 and Pat 11 vs single patient
Ncontrol

R_Ncontrol_vs_AD_woPat4Pat11 = []


dataset_AD_woPat4_Pat11.append(pd.read_excel(
    "Density_matrix_AD_woPat4_woPat11.xlsx"))

for i in range(12):

    tempR = []
    tempR.append(pearson_coeff(
        dataset_AD_woPat4_Pat11[0], dataset_Ncontrol[i]))
    R_Ncontrol_vs_AD_woPat4Pat11.append(tempR)

R_Ncontrol_vs_AD_woPat4Pat11 = np.array(R_Ncontrol_vs_AD_woPat4Pat11)

np.savetxt('pearson_coefficient_Ncontrol_vs_AD_woPat4Pat11.csv',
           R_Ncontrol_vs_AD_woPat4Pat11, delimiter=',')


#Compare common dataset AD without patient 4 and 11 vs single patient NAD

R_NAD_vs_AD_woPat4Pat11 = []


for l in range(7):

    dataset_AD_woPat4_Pat11_woN.append(pd.read_excel(
"Density_matrix_AD_woPat4_woPat11_woPat{}.xlsx".format(patients_AD_woPat4Pa
t11[l])))
    dataset_NAD_woPat4Pat11.append(pd.read_excel(
"Density_matrix_patient{}.xlsx".format(patients_AD_woPat4Pat11[l])))
    tempR = []
    tempR.append(pearson_coeff(
        dataset_AD_woPat4_Pat11_woN[l], dataset_NAD_woPat4Pat11[l]))
    R_NAD_vs_AD_woPat4Pat11.append(tempR)

R_NAD_vs_AD_woPat4Pat11 = np.array(R_NAD_vs_AD_woPat4Pat11)

np.savetxt('pearson_coefficient_NAD_vs_AD_woPat4Pat11.csv',
          R_NAD_vs_AD_woPat4Pat11, delimiter=',')
```
